# The implication of a diversity of non-neuronal cells in disorders affecting brain networks

**DOI:** 10.3389/fncel.2022.1015556

**Published:** 2022-11-11

**Authors:** Micaël Carrier, Kira Dolhan, Bianca Caroline Bobotis, Michèle Desjardins, Marie-Ève Tremblay

**Affiliations:** ^1^Neurosciences Axis, Centre de Recherche du CHU de Québec, Université Laval, Québec City, QC, Canada; ^2^Division of Medical Sciences, University of Victoria, Victoria, BC, Canada; ^3^Department of Psychology, University of Victoria, Victoria, BC, Canada; ^4^Department of Biology, University of Victoria, Victoria, BC, Canada; ^5^Department of Physics, Physical Engineering and Optics, Université Laval, Québec City, QC, Canada; ^6^Oncology Axis, Centre de Recherche du CHU de Québec, Université Laval, Québec City, QC, Canada; ^7^Department of Neurology and Neurosurgery, McGill University, Montreal, QC, Canada; ^8^Department of Molecular Medicine, Université Laval, Québec City, QC, Canada; ^9^Department of Biochemistry and Molecular Biology, The University of British Columbia, Vancouver, BC, Canada

**Keywords:** neurons, synapses, structural and functional connectivity, microglia, astrocytes, oligodendrocytes, vasculature

## Abstract

In the central nervous system (CNS) neurons are classically considered the functional unit of the brain. Analysis of the physical connections and co-activation of neurons, referred to as structural and functional connectivity, respectively, is a metric used to understand their interplay at a higher level. A myriad of glial cell types throughout the brain composed of microglia, astrocytes and oligodendrocytes are key players in the maintenance and regulation of neuronal network dynamics. Microglia are the central immune cells of the CNS, able to affect neuronal populations in number and connectivity, allowing for maturation and plasticity of the CNS. Microglia and astrocytes are part of the neurovascular unit, and together they are essential to protect and supply nutrients to the CNS. Oligodendrocytes are known for their canonical role in axonal myelination, but also contribute, with microglia and astrocytes, to CNS energy metabolism. Glial cells can achieve this variety of roles because of their heterogeneous populations comprised of different states. The neuroglial relationship can be compromised in various manners in case of pathologies affecting development and plasticity of the CNS, but also consciousness and mood. This review covers structural and functional connectivity alterations in schizophrenia, major depressive disorder, and disorder of consciousness, as well as their correlation with vascular connectivity. These networks are further explored at the cellular scale by integrating the role of glial cell diversity across the CNS to explain how these networks are affected in pathology.

## Introduction

The central nervous system (CNS) contains a myriad of neurons, all of which converge in function to support informational exchange (Purves, 2005; Thivierge, 2008; Bandler et al., 2022). This informational exchange between neuronal ensembles can be described *via* CNS networks – which can be classified as functional, structural, or vascular (Sporns, 2013; Bright et al., 2020). The CNS functional connectivity (FC) is defined as the exchange of information in the context of a certain task or CNS state, which is based on the temporal correlation between signals among distinct brain regions (Hampson et al., 2002). For example, previous studies have estimated the topology of information flow in healthy participants at rest (Wang et al., 2017) or performing working memory tasks (Funahashi, 2017), and in patients suffering from locked-in syndrome (Roquet et al., 2016). Structural connectivity (SC), by contrast, exists independent of a given CNS state or functional task, and refers to the anatomical connections between CNS regions (Hagmann et al., 2010; Wang et al., 2011; Tsang et al., 2017). These structural connections are formed *via* axonal white matter tracts, which facilitate the flow of information from the neuronal cell body to the axon terminals, and ultimately to post-synaptic cells (Sporns, 2013). Brain development can be considered a preprogramme important for proper maturation, both structurally and functionally (King et al., 2020). Alteration in this neurodevelopment plan can be detrimental and lead to neurodevelopmental disorder-like behaviors in animal models (Armstrong et al., 2020). FC and SC are tightly interrelated and thus often share similarities. However, they can sometimes diverge in important ways, thereby offering uniquely informative measures of CNS networks (Uddin, 2013). Vascular connectivity (VC) further refers to the topology of blood vessels in the CNS which support the metabolic demands of cellular activity (Shaw et al., 2021). VC is closely tied to FC by means of neurovascular coupling (Bright et al., 2020). A technique often used for measuring FC, functional magnetic resonance imaging (fMRI), evaluates the hemodynamic response rather than neuronal activity directly, making it a paired measure of FC and VC.

**FIGURE 1:**
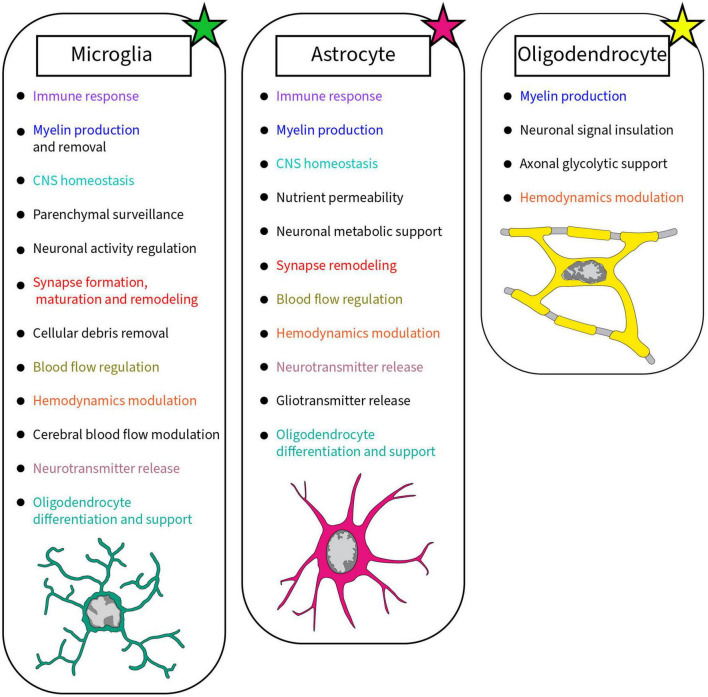
Summary of the roles accomplished by glial cells in the brain discussed in this review. Each glial cell, microglia, astrocyte, and oligodendrocyte, achieves crucial roles in the healthy developing and mature brain. Colors were used to highlight the similar roles between glial cells.

Canonical classification of neurons divides them by the neurotransmitter they use, but varied neuronal populations can also be identified by their neurotrophic, beneficial for neuronal growth, or neuroprotective factors (Que et al., 2019; Sugino et al., 2019; Cizeron et al., 2020). Neurons and non-neuronal cells work together to improve plasticity and enhance the neuronal network by keeping efficient connections (Tremblay, 2020). To ensure proper sending and receiving of neuronal information, the CNS comprises a complex support system primary composed of glial cells such as microglia, astrocytes, and oligodendrocytes achieving various roles (Figure 1; Oberheim et al., 2012; Butt and Verkhratsky, 2018; Foerster et al., 2019; Stratoulias et al., 2019). Microglia, astrocytes and oligodendrocytes were shown to display a wide diversity across the CNS, by which they play main roles as immune sentinels, metabolic regulators and myelin producers, respectively (Davalos et al., 2005; Nimmerjahn et al., 2005; Foerster et al., 2019; Zampieri and Costa, 2022). Even though myelination is mainly performed by oligodendrocytes, microglia are able to help by removing myelin through phagocytosis, while releasing sulfatide, a myelin-specific galactolipid, able to promote myelin basic protein (MBP) production (Gitik et al., 2011; Traiffort et al., 2020). Astrocytes also contribute to the myelination process, similar to microglia, releasing growth factors that influence oligodendrocyte’s maturation (Mason et al., 2001; Traiffort et al., 2020). Recent discoveries highlight the diversity of microglia and astrocytes, as they are highly dynamic cells with states that change rapidly across space and time. Microglial dynamism allows their maintenance of the CNS homeostasis, surveillance of the parenchyma, regulation of neuronal activity and synaptic plasticity, as well as removal of cellular debris (Ginhoux et al., 2010; Matcovitch-Natan et al., 2016; Badimon et al., 2020). While microglia reside in the CNS parenchyma, peripheral immune cells are not necessarily limited by the blood stream, plenty of these cells are able to migrate to the CNS whenever needed (Fani Maleki and Rivest, 2019). The communication between the CNS and peripheral immune system is bidirectional (Watkins and Maier, 1999; Carrier et al., 2021). The recruitment of lymphocytes and monocytes can be triggered by neurotransmitters and neurochemicals released from neurons or *via* cytokines (Kerage et al., 2019). In order to migrate to the CNS during pathology and perform their immune function, these peripheral cells can cross the BBB or take an alternative route through the meninges or choroid plexus (Benakis et al., 2018; Nishihara et al., 2020; Huang et al., 2021). To understand CNS function, looking at structure by investigating the neurovascular unit (NVU) and the synapse can be insightful. This is also relevant in pathology, where functional alterations are associated with observable changes in structure, such as in developmental, emotional and consciousness disorders (Tables 1–3).

**TABLE 1:** Summary of the cellular mechanisms found in schizophrenia and their suggested effect on the brain.

Schizophrenia (SCZ)	
**Cellular mechanisms**	**Hypothesized effect on brain networks**
Impaired astrocytic expression of GMR3 and GABBR1 (Goudriaan et al., 2014).	Whole-brain FC abnormalities (Lynall et al., 2010; Damaraju et al., 2014).
Abnormal lipid metabolism and oxidation-reduction genes in oligodendrocytes (Goudriaan et al., 2014).	Damage to white matter projections, accelerated aging, and reduced SC (Kochunov et al., 2013; Goudriaan et al., 2014) could help account for abnormal cerebral asymmetry (Mitchell and Crow, 2005; Chang et al., 2015).
Oligodendrocyte precursors not exiting the cell cycle (Kerns et al., 2010).	Unmyelinated axons and thus 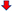 SC (Kelly et al., 2018).
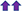 synaptic pruning by microglia (Sekar et al., 2016; Sellgren et al., 2019; Park et al., 2020).	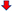 SC and FC could help account for hypoconnectivity between sensory regions and impairments sustaining strong, large-scale connections, (Lynall et al., 2010; Damaraju et al., 2014)
Dysregulated astrocytic release of synaptic remodeling factors (Watanabe et al., 2007).	
Bias towards expression of proteins which inhibit remodeling of the extracellular matrix and brain vasculature (Jeffries et al., 2018)	Impaired and static VC (Won et al., 2013).‘
Abnormal expression of genes associated with GABAergic neuronal migration and GABA signaling in periventricular endothelial cells (Won et al., 2013)	Abnormalities in VC and inhibitory FC/SC (Friston and Frith, 1995; Friston, 1996; Won et al., 2013; Carrier et al., 2020).

**TABLE 2:** Summary of the cellular mechanisms found in disorder of consciousness and their suggested effect on the brain.

Disorder of consciousness (DOC)	
**Cellular mechanisms**	**Hypothesized effect on brain networks**
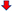 AMPK signaling and, in turn, lactate production by astrocytes and microglia (Saito et al., 2019; Muraleedharan et al., 2020; Monsorno et al., 2022).	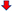 whole-brain FC (Moruzzi and Magoun, 1949; Soddu et al., 2009; Rosazza et al., 2016; Tanabe et al., 2020).
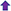 microglial phagocytic activity, contact with neurons (“satellite microglia”), secretion of pro-inflammatory cytokines, and synaptic pruning (Ramlackhansingh et al., 2011; Donat et al., 2017; Krukowski et al., 2021).	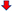 SC, as indexed by increased NFL levels (Adams et al., 2000; Guldenmund et al., 2016; Bagnato et al., 2017, 2021).
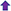 microglial phagocytosis of astrocytic endfeet and thus loss of BBB integrity (van Vliet et al., 2020; Heithoff et al., 2021).	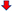 VC (Haruwaka et al., 2019; Heithoff et al., 2021).
Hyperactivity of microglial and astrocytic removal of amyloid-β from cerebrospinal fluid (Bagnato et al., 2018; Wong et al., 2021).	Unknown network implications (Bagnato et al., 2017a, Bagnato et al., 2018).
Impaired blood flow and metabolism in the DMN (Rosazza et al., 2016; Chennu et al., 2017; Zhang et al., 2018).	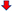 FC and VC in the DMN (Vanhaudenhuyse et al., 2010; Fernández-Espejo et al., 2012; van Vliet et al., 2020).

**TABLE 3:** Summary of the cellular mechanisms found in major depressive disorder and their suggested effect on the brain.

Major depressive disorder (MDD)	
**Cellular mechanisms**	**Hypothesized effect on brain networks**
Altered astrocytic and microglial expression of various glutamate receptors, GABA receptors, gap junction connexin proteins, and glutamate transporters (Choudary et al., 2005; Bernard et al., 2011; Medina et al., 2016).	Abnormal FC in networks involving the hippocampus, locus coeruleus, anterior cingulate cortex, and prefrontal cortex (Kaiser et al., 2016; Wise et al., 2017; Li et al., 2018; Yan et al., 2019).
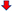 oligodendrocyte density in the amygdala (Hamidi et al., 2004).	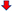 intrinsic FC in the amygdala (Ramasubbu et al., 2014) and SC in networks involving the amygdala (Fang et al., 2012; Coloigner et al., 2019).
Shorter telomeres and 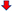 expression of oxidative defense enzymes in oligodendrocytes (Szebeni et al., 2014).	Damage to white matter projections, accelerated aging, and 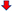 SC (Fang et al., 2012; de Kwaasteniet et al., 2013; Coloigner et al., 2019).
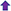 microglial expression of proinflammatory cytokines (Dowlati et al., 2010; Barnes et al., 2017; Wang et al., 2019).	Dysregulating long term potentiation and depression (Innes et al., 2019), thus impairing FC and SC (Fang et al., 2012; de Kwaasteniet et al., 2013; Kaiser et al., 2016; Wise et al., 2017; Li et al., 2018; Coloigner et al., 2019).
Low expression of connexin 30 and 34 in the frontal cortex, mediodorsal thalamus, and caudate nucleus of MDD patients who completed suicide (Ernst et al., 2011; Nagy et al., 2017).	Dysfunctional astrocyte-to-astrocyte signaling, astrocyte-to-oligodendrocyte signaling, FC, and SC in frontoparietal networks (Fang et al., 2012; de Kwaasteniet et al., 2013; Long et al., 2015; Coloigner et al., 2019; Wu et al., 2019).
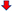 connexin 30 expression in oligodendrocytes of the anterior cingulate cortex in MDD suicide victims (Tanti et al., 2019).	
Ischemic lesions (Alexopoulos et al., 1997; Menard et al., 2017) and white matter lesions (de Groot et al., 2000; Kumar et al., 2000; Taylor et al., 2013; Rushia et al., 2020), especially in the prefrontal cortex.	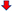 VC, SC, and FC in the frontal cortex and DMN (Coffey et al., 1988; Wu et al., 2011).

## Cellular variety forming the neurovascular unit

To provide a selective border between the CNS and circulatory system, the BBB is formed of a complex NVU (Coelho-Santos et al., 2021). The endothelial cells lie on a basement membrane (Coelho-Santos and Shih, 2020). This main component is wrapped around by pericytes able to control the blood flow (Gonzales et al., 2020). The NVU is the perfect example of glial cell cross-communication, with both microglia and astrocytes shown to play a role in the regulation of blood flow (Mulligan and MacVicar, 2004; MacVicar and Newman, 2015; Mishra et al., 2016; Kleinberger et al., 2017; Császár et al., 2022). Similarly, oligodendrocytes and microglia contribute to the NVU, working in conjunction with astrocytes and endothelial cells to mediate hemodynamics (Rajani and Williams, 2017; Haruwaka et al., 2019; Kugler et al., 2021). Astrocytes achieve this function using their astrocytic end-feet arranged around the outer layer of the NVU and are able to constrict the vasculature (Mulligan and MacVicar, 2004). Microglia, newly identified actors of the NVU, also secrete factors such as nitric oxide and cyclic GMP contributing to cerebral blood flow control (Lacoste and Gu, 2015; Császár et al., 2022). They additionally have a more direct role, being part of the outermost layer of the parenchyma or *glia limitans*, interacting closely with the other parenchymal cells (Bisht et al., 2016; Joost et al., 2019; Carrier et al., 2020). The NVU is a crucial element of the CNS, able to provide the nutrients and oxygen required by the CNS while directly coordinating their delivery with the local brain activity (Carrier et al., 2020). Neuronal activity involves a complex system metabolically that recruits the brain community with major influences from all glial cell types, dynamically changing together CNS connectivity on molecular and cellular levels (Tremblay, 2011; Schafer et al., 2013; Chung et al., 2015; Hughes and Appel, 2019; Lee et al., 2021a).

## Non-neuronal cellular diversity interacting with neurons and synapses

Microglia, the specialized immune cell population of the brain, interact with neurons in many ways (Marinelli et al., 2019). Microglial recruitment is notably performed by neurons through soluble and membrane bound factors. For instance, the neuron-derived fractalkine (CX3CL1) binds to CX3CR1, which is largely found on microglia (Paolicelli et al., 2011; Carrier et al., 2021). Furthermore, the absence of this fractalkine signaling pathway is shown to lead to social impairments (Cordella et al., 2021). Perturbations in the CNS microenvironment including levels of peptides and neurotransmitters are also sensed by microglia notably through purinergic signaling *via* P2Y12 receptor (Chen et al., 2019). Another role of microglia involving their relationships with neurons and synapses is synaptic pruning (Lewis, 2021). As phagocytes, microglia can remove excessive or weak network connections (Geloso and D’Ambrosi, 2021). Among the underlying mechanisms, microglia can partially engulf pre-synaptic elements *via* trogocytosis, effectively nibbling the synapse (Weinhard et al., 2018). Microglia are further able to rewire networks in a non-phagocytic way in a process called synaptic stripping where they physically separate the pre- and post-synaptic elements (Kettenmann et al., 2013).

Astrocytes additionally play crucial roles in communication with neurons. For instance, astrocytes maintain extracellular homeostasis, nutrient permeability and provide metabolic support to neurons in the CNS (Verkhratsky and Nedergaard, 2018; Kim et al., 2019; Siracusa et al., 2019). Astrocytes control Ca^2+^ variations and, like microglia, they hold plenty of K^+^ channels which further influence neurotransmitters release (Madry et al., 2018; Turovsky et al., 2020). Regarding synaptic function, astrocytes and microglia enhance synaptic sites, *via* direct contact or support by soluble factors, proposing the concept of “quad-partite synapses” comprised of pre and postsynaptic neurons plus astrocytes and microglia, allowing for the maintenance of homeostasis when a neurotransmitter also binds to the adjacent astrocyte and microglia to modulate different pathways (Tremblay and Majewska, 2011; Farhy-Tselnicker and Allen, 2018; Kim et al., 2020). Moreover, astrocytes are associated with synaptic pruning, together with microglia, being able to engulf synaptic elements notably *via* the MEGF10, MERTK, classical complement and TREM2 pathways (Barnum, 1995; Lee and Chung, 2019). Oligodendrocytes, on their part, are distant contributors of the quad-partite synapse as insulator of the neuronal signal (Miyata, 2019; Carrier et al., 2020). Oligodendrocytes’ membranes wrap around axonal tracts, forming a sheath which facilitates saltatory conduction (Duncan et al., 2021). Myelin can cover up to 60 axons per oligodendrocyte, depending on the CNS parenchymal location (Kuhn et al., 2019; Swire et al., 2021). A lot of energy and metabolic functions are involved in producing myelin proteins, such as MBP and myelin proteolipid protein (PLP) (Kuhn et al., 2019). In addition, myelin acts as a cover, avoiding the diffusion of metabolites through the axon. Instead, oligodendrocytes can provide glycolytic products to the axon, due to the presence of monocarboxylate transporter (MCT-1) (Duncan et al., 2021). Oligodendrocytes also support neurons *via* cytosolic myelin channels, able to bidirectionally transport macromolecules between the oligodendrocyte process and the axon (Simons and Nave, 2016; Frühbeis et al., 2020). Large evidence highlights the implication of microglia and astrocytes in roles attributed to oligodendrocytes (Domingues et al., 2016; Lombardi et al., 2019; Traiffort et al., 2020; Kalafatakis and Karagogeos, 2021; Santos and Fields, 2021). Microglia are able to phagocytose myelin to prevent excessive myelin production in zebrafish (Hughes and Appel, 2020). Furthermore, microglia and astrocytes both secrete trophic factors which support the differentiation of oligodendrocyte progenitor cells, as extensively reviewed in Traiffort et al. (2020). Microglia can also take an active role by pruning the oligodendrocytic lineage cells directly to regulate the myelination process (Nemes-Baran et al., 2020). Therefore, glial cells partake in neuronal networks function in many ways, helping one another, and acting on both the gray and white matter. However, microglial interactions with neurons can become altered, leading to disorders (Carrier et al., 2021).

Immune and glial cells, which play beneficial roles during development and plasticity, also contribute to recovery during pathological processes (Skaper et al., 2018; Han et al., 2021). However, these critical roles in healing can become affected by genetic and environmental factors, for example causing prolonged inflammation, hence leading to various disorders depending on the stage of life (Comer et al., 2020a,b; Carrier et al., 2021). These unresolved issues can leave the CNS in an unhealthy state, due to neuronal circuit alterations, increasing the risk of disorders such as schizophrenia (SCZ) and major depressive disorder (MDD) (Stevenson et al., 2020) and can also potentially exacerbate disorders of consciousness (DOCs) (Bagnato et al., 2021). DOCs here refer to a state of consciousness defined by impaired wakefulness (e.g., inability to receive sensory stimuli) and awareness (of oneself and/or the environment) after brain damage. In the next sections, we will examine how the structural, functional and vascular connectivity of the CNS is affected in these disorders, then go deeper into the glial dynamics at play in developmental (focusing on SCZ as an example), consciousness (DOCs) and mood (particularly MDD) disorders.

## Glial cell remodeling of neural and vascular networks in schizophrenia

### Functional connectivity

Schizophrenia has been robustly associated with altered brain FC (Lynall et al., 2010; Damaraju et al., 2014; Lei et al., 2020); so much so that SCZ has long been considered a “disconnection syndrome” (Friston and Frith, 1995; Friston, 1996; Lei et al., 2020). When measuring resting state FC *via* fMRI, patients with SCZ show long-term hyperconnectivity between the thalamus and sensory cortices, and a contrasting hypoconnectivity between cortical sensory regions (Damaraju et al., 2014). Dynamic FC measurements suggest that patients with SCZ have impairments in sustaining strong, large-scale connections, while diverse connectivity patterns instead arise (Lynall et al., 2010; Damaraju et al., 2014). These findings were corroborated by studying a large cohort of patients with SCZ, as whole-brain FC measured *via* fMRI could discriminate, using machine learning algorithms, patients from controls with an average accuracy of 81% (Lei et al., 2020). Whole-brain FC measures were most informative of the individual categorization; however, the thalamus and temporal cortex were primary contributors to this overall dysconnectivity (Lei et al., 2020).

Since glial cells perform critical functions in synaptic transmission and network development, their role in “disconnection syndromes” is beginning to be investigated (Damaraju et al., 2014; Fields et al., 2015; Dietz et al., 2020). For example, using genome-wide association analyses on human data, SCZ was associated with abnormal astrocytic and oligodendrocytic, but not microglial, genes (Goudriaan et al., 2014). The affected astrocyte genes notably encoded GMR3 and GABBR1: G-protein coupled receptors for glutamate and GABA, respectively, which enable astrocytes to detect neurotransmitters and respond by releasing gliotransmitters, thereby altering quad-partite synaptic strength (Goudriaan et al., 2014). Since glutamate and GABA are used throughout the mammalian brain (Zhou and Danbolt, 2013), this finding could help explain the brain-wide changes of FC observed in SCZ (Lei et al., 2020). Thus, genetic abnormalities in patients with SCZ have large implications for overall FC *via* altering synaptic formation, efficacy, and plasticity (Goudriaan et al., 2014).

### Structural connectivity

The disconnection hypothesis of SCZ etiology includes SC abnormalities (Friston, 1996; Fields et al., 2015). For instance, SCZ symptomology is hypothesized to be, at least in part, the resultant of disrupted interhemispheric communication (Rotarska-Jagiela et al., 2008; Guo et al., 2013a; Chang et al., 2015). Hemispheric asymmetry is observed ubiquitously in both vertebrates and invertebrates, as it is thought to facilitate functional specialization of brain networks (e.g., language networks are typically lateralized to the left hemisphere; (Corballis, 2014)). Abnormal hemispheric asymmetry has been associated with SCZ — specifically in language networks, as there is reduced left cerebral hemisphere dominance (Mitchell and Crow, 2005; Chang et al., 2015). In fact, reduced left hemisphere lateralization in the temporal lobes has been correlated with SCZ symptom severity: patients with reduced gray matter volume and hemodynamic activity, the variation of blood movement in the vasculature, in the left temporal lobe tended to experience more severe auditory hallucinations (Oertel et al., 2010). A study of 1,963 patients revealed that brain-wide, rather than regional, SC was most severely impaired in SCZ (Kelly et al., 2018). For example, it was reported that the major white matter fasciculi of patients with SCZ had significantly decreased SC, as indicated by reduced fractional anisotropy in diffusion tensor imaging (DTI) data (Kelly et al., 2018). Fractional anisotropy is a measure of water diffusion through the brain, with high values representing restricted diffusion largely in one direction (this is inferred to be the result of white matter tracts) and low values indexing a free flowing diffusion (which is inferred to represent a reduction in white matter volume and/or integrity (Alexander et al., 2007)). Thus, the SC and FC findings in patients with SCZ mirror each other: there does not seem to be selected foci which can account for the dysconnectivity observed, rather, SC and FC abnormalities appear widespread in the SCZ brain (Damaraju et al., 2014; Kelly et al., 2018; Lei et al., 2020). Importantly, this global white matter degradation may be correlated with accelerated biological aging, and thus cognitive decline, in patients with SCZ (Kochunov et al., 2013). When whole-brain averaged fractional anisotropy is used as a biomarker for age-related changes in SC, past literature has found a significant interaction between biological age and SCZ symptoms (Kochunov et al., 2013).

As for the mechanisms involved in the decreased SC observed in SCZ, many studies are pointing to abnormalities in oligodendrocytes (Kerns et al., 2010; Goudriaan et al., 2014). When studying human data *via* genome-wide association analyses, SCZ was linked to abnormal oligodendrocyte gene sets which regulate lipid metabolism and gene transcription (Goudriaan et al., 2014). Furthermore, abnormalities were observed in oligodendrocyte oxidation-reduction gene sets, which would affect lipid metabolism and could lead to the production of reactive oxygen species, thereby damaging the myelin sheath *via* oxidative stress (Goudriaan et al., 2014). Thus, disrupted oligodendrocyte lipid metabolism may account for the decreased white matter integrity observed in SC studies on SCZ patients (Kochunov et al., 2013; Goudriaan et al., 2014), and may also help explain the accelerated biological aging observed in SCZ (Kochunov et al., 2013; Carrier et al., 2021). Further, white matter degeneration at the level of commissural fibers may help explain the reduced left cerebral hemisphere dominance robustly observed in SCZ (Mitchell and Crow, 2005; Rotarska-Jagiela et al., 2008; Guo et al., 2013a; Chang et al., 2015). Another study used polymerase chain reaction in *post mortem* human brain samples to examine mRNA expression of genes associated with different stages of the cell cycle (Kerns et al., 2010). It was found that, in SCZ, oligodendrocytes do not properly mature and myelinate neurons, as oligodendrocyte precursors in the SCZ brain avoid exiting the cell cycle (Kerns et al., 2010). The expression of genes associated with maintenance of the cell cycle was increased in patients with SCZ relative to controls, while gene expression associated with cell cycle arrest was decreased in SCZ (Kerns et al., 2010). Thus, cells of the oligodendrocytic lineage appear to face a myriad of challenges in the brain of patient with SCZ: accelerated aging (Kochunov et al., 2013), oxidative damage (Goudriaan et al., 2014), disrupted lipid metabolism (Goudriaan et al., 2014), and a reduced maturation toward myelinating oligodendrocytes (Kerns et al., 2010). Presumably this accelerated aging and oxidative damage would have a maladaptive effect on microglia (e.g., a hyperactive proinflammatory response, increased cytokine release, reduced expression of neuroprotective factors (Gillen et al., 1981; Niraula et al., 2017)) which warrants further investigation.

In addition to oligodendrocytes, the astrocytic and microglial cell populations influence SC by means of their synaptic pruning capabilities (Kuijlaars et al., 2016; Sellgren et al., 2019; Park et al., 2020). For instance, using cultures of human patient-derived microglia-like cells as a model of synaptic pruning, an increased phagocytosis of synaptic elements was found in the SCZ patient-derived cells, relative to a population of cells from healthy controls (Sellgren et al., 2019). Further, this same study found that exposing cultures to minocycline, an antibiotic which normalizes microglial proinflammatory and synaptic pruning functions under certain contexts, reduced microglia-mediated synapse uptake (Scott et al., 2018; Sellgren et al., 2019; Celorrio et al., 2022). Thus, microglia have shown promise as therapeutic targets to slow the neurogenerative changes observed in SCZ, particularly as a pre-emptive measure for individuals identified as at risk of developing SCZ (Glausier and Lewis, 2013; Cannon, 2015; Sellgren et al., 2019). Indeed, this hypothesis and proposed treatment corroborate longitudinal studies of SCZ: synaptic pruning in the cerebral cortex is prevalent in late adolescence and early adulthood, which coincides with the period when SCZ symptoms typically begin to arise (Petanjek et al., 2011; Cannon, 2015). Further, mutations in the complement component 4 gene constitute a risk factor for SCZ development (Sekar et al., 2016), and notably, this SCZ susceptibility gene regulates microglia-mediated synaptic pruning in mice during early postnatal development (Sekar et al., 2016; Sellgren et al., 2019; Yilmaz et al., 2021). Similarly, a study of cultured cerebral interneurons derived from patients with SCZ or healthy controls found that, in both types of cultures, exposure to inflammatory-cytokine-releasing microglia resulted in reduced interneuron arborization and synapse formation (Park et al., 2020). Critically, however, once the microglia were removed from the cell cultures, the neurons derived from healthy controls began to recover, whilst the SCZ patient-derived cells did not (Park et al., 2020). As for astrocytes, they secrete synaptic remodeling factors including fatty acid binding proteins (FABP7), a protein which has tentatively been associated with SCZ development in mice and humans (Watanabe et al., 2007). It is worth noting, however, that some studies have failed to replicate this FABP7 and SCZ association (Iwayama et al., 2010). One should note that as longitudinal studies are scarce, it is a possibility that the myelin decrease seen would be in fact a lack of myelination happening during development.

### Vascular connectivity

Abnormalities in vascular and blood protein networks were also identified in SCZ (Jeffries et al., 2018). Correlative models predicting the potential development of SCZ in humans were developed, revealing that blood factors differentiating patients at risk for SCZ from controls largely involve proteins that regulate tissue remodeling (e.g., of the BBB) (Jeffries et al., 2018). Control subjects showed greater co-expression of proteins exerting complementary effects on CNS remodeling, presumably to facilitate homeostasis (Jeffries et al., 2018). For example, controls demonstrated elevated co-expression of plasminogen activator inhibitor-1 and several metalloproteinases: proteins which inhibit and promote vascular remodeling, respectively. In contrast, those at risk for SCZ, and especially patients who eventually developed SCZ, showed co-expression of proteins which inhibited remodeling of the extracellular matrix and brain vasculature (Jeffries et al., 2018). Thus, the findings of Jeffries et al. (2018) suggest that SCZ etiology may be inversely related to the capacity of brain vascular networks to dynamically remodel (Won et al., 2013). That is, endothelial cells in the neurovascular network largely influence the development of neural networks, including later established FC and SC (Won et al., 2013; Andreone et al., 2015). Endothelial cells, in both pial and periventricular blood vessels, exert chemoattractant functions *via* their expression of GABA_*A*_ receptors and secretion of GABA (Won et al., 2013). These properties facilitate a bidirectional communication between endothelial cells and GABAergic neurons, thereby promoting neuronal migration to specific locations of the developing cortex (Won et al., 2013). Critically, it was found that many genes associated with SCZ are upregulated in periventricular endothelial cells (Won et al., 2013). These genes modulate GABAergic neuronal migration and GABA signaling, thus providing a potential mechanism for the abnormal GABAergic FC often observed in SCZ (Won et al., 2013; Goudriaan et al., 2014; Hoftman et al., 2015). Abnormalities in the vascular network during embryonic and postnatal development may therefore place individuals at risk for developing SCZ later in life, and could help explain the etiology of this neurodevelopmental disorder (Won et al., 2013; Andreone et al., 2015; Carrier et al., 2020). Glial cells are of great importance in regulating brain vasculature: for example, astrocytic endfeet regulate vasoconstriction and vasodilation (MacVicar and Newman, 2015), as well as BBB permeability (Abbott et al., 2006). However, the precise mechanisms by which glial cells influence vascular brain networks, and how these mechanisms can be altered in SCZ, requires further investigation: for example, microglial-vascular interactions are only beginning to receive attention in the literature (Bisht et al., 2021; Kisler et al., 2021).

In summary (Table 1), glial cells play many important roles in establishing and maintaining network dynamics on the levels of functional, structural, and vascular connectivity (Won et al., 2013; Goudriaan et al., 2014; Andreone et al., 2015; Kelly et al., 2018; Carrier et al., 2020). Aberrant functioning of these mechanisms can help explain some of the etiology and symptomology underlying SCZ, as well as suggest potential modes for treatment (Bernstein et al., 2009; Takahashi and Sakurai, 2013; Bisht et al., 2021). However, glial cells are implicated in a wide variety of brain diseases and their influence is not restricted to neurodevelopmental disorders alone. In the next section, we will overview non-neuronal cell contributions to the brain networks underlying consciousness.

## Anesthesia/disorders of consciousness: Recovery of consciousness

### Functional connectivity

In a clinical context, consciousness is defined by two variables: (1) awareness and (2) wakefulness (Gosseries et al., 2011; Mecarelli et al., 2019). Chronically reduced levels of awareness and/or wakefulness thus define a DOC; for example, a comatose patient (Gosseries et al., 2011). The same can be said for anesthesia-induced consciousness, referring to a controlled and medically induced coma (Brown et al., 2010; An et al., 2015). As for the relevant FC, previous literature revealed a robust relationship between conscious perception and frontoparietal network activation (Linden et al., 1999; Corbetta and Shulman, 2002; Corbetta et al., 2008; Braga et al., 2017); specifically, the dorsal and ventral attention networks (the DAN and VAN, respectively) (Vossel et al., 2014). As for their functions, the DAN mediates visuospatial attention (e.g., when using a stimulus to direct a participant’s attention to one side of a screen) (Vossel et al., 2014). In contrast, the VAN has been shown to mediate attention when behaviourally relevant stimuli occur unexpectedly (e.g., during an oddball paradigm) (Vossel et al., 2014). “Conscious awareness” (defined by the ability of an individual to report their perception of a stimulus) is linked to greater connectivity in these frontoparietal attention networks (Linden et al., 1999; Corbetta and Shulman, 2002; Gosseries et al., 2011). This effect has been replicated for participants in various conscious states: including typical wakefulness, DOCs, during sleep, and under anesthesia (Tanabe et al., 2020). The second aforementioned tenet of consciousness–neurological arousal–is defined by an organism’s ability to respond to information in a context-specific and appropriate manner (Gosseries et al., 2011). It has been long known that the reticular activating system (RAS) is key to arousal (Moruzzi and Magoun, 1949), while functional abnormalities in the RAS cause brain disorders (Garcia-Rill, 1997). In fact, the connectivity of the RAS has been shown to inversely correlate with DOC severity (Mecarelli et al., 2019). As the name suggests, this brain network originates in the reticular formation, extends up the midbrain, and into the thalamus, from which it coordinates global cortical activity (Moruzzi and Magoun, 1949).

Many neurotransmitters critical to the functioning of the RAS, general anesthetics (e.g., sevoflurane), and substances accelerating anesthesia emergence (e.g., caffeine) stimulate adenosine monophosphate-activated protein kinases (AMP) (Finley, 2019). Thus, AMPK pathways appear as a critical mechanism underlying the FC by facilitating awareness and wakefulness. Importantly, AMPK is found throughout the mammalian brain (e.g., in the thalamus, hypothalamus, cortical pyramidal neurons), making the kinase a great target for modulating widespread frontoparietal and RAS brain networks (Finley, 2019). To track metabolic pathways, proton (^1^H) magnetic resonance spectroscopy can be used to monitor glucose flow through the brain, while ^13^C glucose mass spectroscopy can be used to monitor a variety of brain metabolites (e.g., lactate, glutamate; Muraleedharan et al., 2020). Said techniques have thus been used in mouse and fly models of brain metabolism, demonstrating that AMPK signaling is highly reliant on glial cells (Muraleedharan et al., 2020). For example, once glutamate is released into the synaptic cleft, astrocytes uptake the neurotransmitter *via* their glutamate transporters (e.g., GLAST and GLT-1), thereby regulating synaptic transmission (Bélanger et al., 2011; Muraleedharan et al., 2020). Glycolysis then occurs within the astrocyte, producing the lactate required by neurons for their own ATP production (Bélanger et al., 2011; Muraleedharan et al., 2020). AMPK activation is critical for this astrocytic lactate production, as spectroscopy and immunohistochemistry data suggest that AMPK knockout mice have impaired lactate production, resulting in neuronal cell death and volume reduction throughout the cerebral cortex during development (Muraleedharan et al., 2020). Much like astrocytes, microglia also produce lactate *via* glycolysis, a process which is again mediated by AMPK signaling (Saito et al., 2019; Monsorno et al., 2022) raising the question if microglia are another potential lactate supplier for neurons. Thus, it appears that part of the mechanism by which anesthesia and DOCs alter FC is by targeting AMPK pathways; thereby depriving neurons of their energy source and resulting in widespread neuronal hypoactivity robustly observed in reduced states of consciousness (Moruzzi and Magoun, 1949; Soddu et al., 2009; Rosazza et al., 2016; Tanabe et al., 2020). FC measured by electroencephalography (EEG) was shown to correlate with frontoparietal glucose metabolism, behavioral responsiveness, and recovery in humans with DOCs (Chennu et al., 2017).

### Structural connectivity

In agreement with the FC literature, SC deficits in patients with DOCs are consistently identified in thalamocortical and frontoparietal networks (Wheeler and Malinak, 1989; Adams et al., 2000; Fernández-Espejo et al., 2012), and in more severe DOC cases, the brainstem (Edlow et al., 2012; Snider et al., 2020). In fact, a DTI study of patients with DOCs found reduced SC in four axonal tracts around the brainstem to be associated with DOC severity; further implicating the RAS network in maintaining wakefulness (Wu et al., 2018). Thalamic neurons play a critical role in the RAS and in establishing long-range cortico-thalamo-cortical connections which are networks thought to be essential for consciousness (Adams et al., 2000; Gosseries et al., 2011; Mecarelli et al., 2019; Tanabe et al., 2020). SC impairments do not tend to be localized, however: widespread reductions in SC, as measured by fractional anisotropy (a metric extracted from DTI measures), have been associated with DOC severity (Adams et al., 2000; Guldenmund et al., 2016; Wu et al., 2018). For example, behavioral measures of DOC severity were shown to inversely correlate with radial diffusivity, contrary to axial diffusivity (Wu et al., 2018). The data suggests that DOC related deficits in SC do not result from axonal degeneration, but are rather caused by demyelination, thereby implicating oligodendrocytes (and their interactions with other non-neuronal cells) in DOC pathology (Wu et al., 2018). This trend of global SC impairment is perhaps unsurprising, as DOCs are the result of major brain damage, either traumatic or non-traumatic (Gosseries et al., 2011; Guldenmund et al., 2016). This would imply the implication of astrocytes and microglia, as both glial types are largely involved in post-brain injury inflammatory responses; for example by releasing various cytokines, chemokines and growth factors (Karve et al., 2016). However, their exact role in DOC pathology remains to be elucidated, as most studies have focused on general traumatic brain injuries (i.e., not necessarily DOC inducing), and the consequences of brain injuries tend to be largely heterogeneous (Goldstein et al., 2010; Karve et al., 2016).

Given the axonal demyelination and brain injury mentioned above, studies have begun to investigate biomarkers of inflammation and tissue damage in patients with DOCs (Bagnato et al., 2017, 2021; Sharma et al., 2020). For example, a biomarker of axonal injury known as neurofilament light chain (NFL) was measured at higher concentrations in the serum and cerebrospinal fluid of patients with DOCs, relative to controls (Bagnato et al., 2017, 2021). These elevated NFL levels may indeed result from hyperactive microglia induced by brain damage, thereby initiating chronic inflammation, and incidentally making the damage worse (Bagnato et al., 2021). Specifically, it is hypothesized that, after DOC inducing brain injury, microglia show increased: phagocytotic activity, contacts with neurons (“satellite microglia”), secretion of pro-inflammatory cytokines, and synaptic pruning (Ramlackhansingh et al., 2011; Donat et al., 2017; Krukowski et al., 2021). As outlined by Bagnato et al. (2021), increased NFL levels may additionally result from a loss of BBB integrity and altered amyloid-β levels, evidence for which have been observed after traumatic brain injury in rats (Mannix and Whalen, 2012; van Vliet et al., 2020; Wong et al., 2021) and in patients with DOCs (Bagnato et al., 2017, 2018). Microglia and astrocytes also play critical roles in maintaining BBB integrity and amyloid-β levels, further implicating these cells in the observed DOC related NFL concentrations (Rogers et al., 2002; Mannix and Whalen, 2012; Ries and Sastre, 2016; Haruwaka et al., 2019; Heithoff et al., 2021). Firstly, microglia and astrocytes have been shown to regulate BBB permeability as discussed above (Abbott et al., 2006; da Fonseca et al., 2014; Haruwaka et al., 2019; Bisht et al., 2021; Heithoff et al., 2021). Under chronic inflammatory conditions, as would be the case in DOCs, microglia engulf astrocytic endfeet *via* phagocytosis, thereby making the BBB more permeable (Haruwaka et al., 2019; Heithoff et al., 2021). This mechanism could help explain the increased NFL levels in patients with DOCs, as causal brain injury combined with reduced BBB integrity would enable NFL to enter the patient’s circulatory system with ease (Bagnato et al., 2021). Furthermore, both microglia and astrocytes play critical roles in the removal of amyloid-β (Rogers et al., 2002; Mannix and Whalen, 2012; Ries and Sastre, 2016), and thus, their hyperactivity could explain reduced amyloid-β levels in the cerebrospinal fluid of patients with DOCs (Bagnato et al., 2018). Glial cells indeed appear to be a key factor in DOC brain connectivity, so much so that clinical trials (Pizzol, 2021) have begun to investigate the effects of minocycline, which normalizes microglial functions but also shows potential in reducing chronic inflammation after traumatic brain injury, in treating DOCs (Scott et al., 2018; Celorrio et al., 2022). If administered acutely after brain injury, the pharmacological effects of minocycline include a reduction in the total number of microglia, and for the microglia that remain, a diminished major histocompatibility complex II expression (Celorrio et al., 2022) suggesting a reduction in microglia-mediated CNS inflammation. This mitigation of the acute proinflammatory actions of microglia may thus serve to promote neuroprotective mechanisms [e.g., by limiting section of pro-inflammatory cytokines and normalizing synaptic pruning; (Ramlackhansingh et al., 2011; Donat et al., 2017; Krukowski et al., 2021)]. Indeed, acute minocycline administration was found to lessen long-term neuronal, white matter, and synaptic degeneration in mice with a traumatic brain injury, relative to animals given a saline vehicle (Celorrio et al., 2022). However, it is important to note that clinical trials on the ability of minocycline to treat SCZ have produced mixed results, likely because minocycline does not exert microglia-specific effects (Möller et al., 2016; Šimončičová et al., 2022). Thus, further research is required to elucidate the detailed mechanisms by which minocycline acts on non-neuronal brain cells and identify therapeutics with more specific targets.

### Vascular connectivity

As highlighted previously, brain injuries often result in increased BBB permeability, which is likely due to changes in microglial and astrocytic function (Abbott et al., 2006; da Fonseca et al., 2014; Haruwaka et al., 2019; Bisht et al., 2021; Heithoff et al., 2021). Thus, abnormalities in the NVU, due to prolonged inflammation after brain injury, are to be expected in DOCs. Impairments of the BBB have been widely studied in the context of general brain injuries (Glushakova et al., 2014; Haruwaka et al., 2019; van Vliet et al., 2020). However, to our knowledge, BBB alterations in brain injuries which specifically cause DOCs remain to be examined. Treatments for DOCs (e.g., spinal cord stimulation) are thought to produce their beneficial effects, at least partly, by stimulating blood flow to the frontal and parietal cortices (Zhang et al., 2018). In addition to blood flow, positron emission tomography (PET) studies suggest that frontoparietal metabolism is impaired in patients with DOC (Rosazza et al., 2016; Chennu et al., 2017). A specific frontoparietal network known as the “default mode network” (DMN) is often associated with altered states of consciousness and refers to the brain regions which show increased hemodynamic activity when a person is not focused on external stimuli (e.g., when daydreaming) (Raichle et al., 2001; Qin and Northoff, 2011). Thus, the DMN represents the pattern of “default” brain activity observed when one is not engaged with the outside world; accordingly, the DMN activity shows a robust negative correlation with activity in the VAN/DAN (Vanhaudenhuyse et al., 2010; Fernández-Espejo et al., 2012; Rosazza et al., 2016; Chennu et al., 2017; Zhang et al., 2018). This DMN often demonstrates reduced blood flow and metabolism in patients with DOCs (Rosazza et al., 2016; Chennu et al., 2017; Zhang et al., 2018), likely reflecting the fact that FC is decreased in the DMN of affected patients (Vanhaudenhuyse et al., 2010; Fernández-Espejo et al., 2012). However, it is worth noting that many of these studies identified DMN areas as *a priori* regions of interest (Rosazza et al., 2016; Chennu et al., 2017), and this may be biasing the general consensus that the DMN is involved (e.g., DOCs may be more accurately described *via* a widespread hypometabolism, but by restricting our search to the DMN, the role of this network becomes hyperbolized (Stender et al., 2014)).

Brain network dynamics are clearly important for the maintenance of behavioral wakefulness and neurological arousal (Edlow et al., 2012; Mecarelli et al., 2019; Snider et al., 2020). Further, they are heavily implicated in “conscious perception”: the ability to access internal mental states (Linden et al., 1999; Corbetta and Shulman, 2002; Corbetta et al., 2008; Braga et al., 2017). Even with current hypotheses (Table 2), further research is required to elucidate the mechanisms by which glial cells contribute to the relevant brain networks: given the roles that non-neuronal cells play in remodeling brain connectivity, there is little doubt that astrocytes, microglia, and oligodendrocytes have an important, yet often overlooked, contribution to the RAS, VAN, DAN, and DMN (Tremblay, 2011; Tremblay and Majewska, 2011; Schafer et al., 2013; Chung et al., 2015; Hughes and Appel, 2019; Duncan et al., 2021; Lee et al., 2021a). As for specific recommendations for future research, it would be informative to investigate human glial cell structure and function in the context of DOC inducing brain injuries using high resolution techniques such as electron microscopy, since most of the current literature is confined to more general traumatic brain injuries (Ramlackhansingh et al., 2011; Glushakova et al., 2014; Haruwaka et al., 2019; van Vliet et al., 2020; Krukowski et al., 2021). This is especially problematic as traumatic brain injuries are very heterogeneous and can have differing effects ranging from chronic loss of consciousness to epilepsy to depression (Goldstein et al., 2010; Karve et al., 2016). Furthermore, advancements in treating DOCs could be made if network neuroscience expanded beyond its typical “neuro-centric” quantification of neural networks and moved into analyzing brain networks more comprehensively. To our knowledge, there has yet to be a study mapping topological networks of quad-partite synapses, or the interactions between neural, vascular, and glial connectivity in altered states of consciousness.

## Major depressive disorder

### Functional connectivity

Major depressive disorder in humans has been associated with characteristic changes of resting-state FC, specifically in frontal cortical regions of the DMN (Kaiser et al., 2016). This network spans the midline of the brain, including the prefrontal cortex, cingulate cortex, precuneus, and inferior parietal cortices (Raichle et al., 2001). The DMN is thought to be associated with self-referential processing and directing attention “inwards” (e.g., through introspection, metacognition) (Raichle et al., 2001; Qin and Northoff, 2011), and it has been implicated in a variety of psychological/brain disorders ranging from MDD to Alzheimer’s disease (Broyd et al., 2009). In fact, studies have correlated abnormal DMN connectivity with the severity of specific MDD symptoms: more dynamic FC (defined by greater standard deviation in resting connectivity over time) between the medial prefrontal cortex and insula was associated with more frequent rumination (Kaiser et al., 2016) as measured *via* the Behavioral Activation for Depression Avoidance Subscale (Kanter et al., 2007). The same study also found MDD severity to positively correlate with more dynamic connectivity between the medial and dorsolateral regions of the prefrontal cortex (Kaiser et al., 2016). General connectivity abnormalities and instability of the DMN is a ubiquitous finding in patients with MDD, which is hypothesized to help explain classic MDD symptoms, such as depressive fixation on self and difficulty engaging with outside activities (Brooks and Lippman, 1985; Wise et al., 2017; Yan et al., 2019; Saris et al., 2020). In addition to the DMN, past research has found robust hypoconnectivity in frontoparietal control networks (e.g., the DAN), which are key for effectively directing one’s attention to environmental stimuli; further explaining why patients with MDD may have difficulty engaging with their external environment (Kaiser et al., 2016; Ye et al., 2016; Li et al., 2018). Reduced interhemispheric resting-state FC between bilaterally symmetrical brain regions was also ubiquitously observed in patients with MDD: for example, in treatment resistant MDD (Guo et al., 2013b), recurrent MDD (Zheng et al., 2022), and first episode drug naive MDD (Wang et al., 2013). The bilateral regions displaying reduced interhemispheric FC, relative to controls, included the medial orbitofrontal gyrus, parahippocampal gyrus, medial prefrontal cortex, fusiform gyrus, and calcarine cortex (Guo et al., 2013b; Wang et al., 2013; Zheng et al., 2022). Finally, abnormalities identified in limbic networks (e.g., involving the amygdala, thalamus, and hippocampus) provide potential mechanisms for the chronic dysphoria and anhedonia commonly experienced in MDD (Kaiser et al., 2016; Ye et al., 2016; Li et al., 2018).

In trying to explain the altered FC observed in patients with MDD, neurobiological studies have noted abnormalities in astrocyte and microglial signaling which facilitate neurochemical communication and restore homeostasis at the synapse (Choudary et al., 2005; Medina et al., 2016). For instance, altered mRNA expression of several glutamate receptors (e.g., *AMPA1, AMPA3, GluR5, GluR-KA2, mGluR5*), GABA receptors (e.g., *GABAARβ3, GABAARδ, GABAARγ2*), gap junction connexin proteins (e.g., *connexin 43* and *30*), and glutamate transporters (e.g., *GLT-1, GLAST*) have been identified in the hippocampus (Medina et al., 2016), locus coeruleus (Bernard et al., 2011), anterior cingulate cortex (Choudary et al., 2005), and left dorsolateral prefrontal cortex (Choudary et al., 2005) of patients diagnosed with MDD. In fact, glia influence neural communication in MDD so strongly that depressive symptoms can be induced in rats *via* selectively ablating astrocytes in the prefrontal cortex (Banasr and Duman, 2008). Histopathological studies of *post mortem* human brain samples have found that, relative to healthy controls, total glial cell density is reduced in the anterior cingulate cortex (Cotter et al., 2001) and dorsolateral prefrontal cortex (Cotter et al., 2002) of patients with MDD. However, glial cell nuclei were analyzed indiscriminately by Cotter et al. (2001, 2002) such that counts of astrocytes, microglia, and oligodendrocytes were combined; thus, we do not know how specific glial subtypes were altered. Many genetic studies have focused on altered mRNA expression in astrocytes, as this glial subtype can form cell-to-cell junctions, creating their own glial communication network to help neurons return to homeostasis after electrochemical activity (Kiyoshi and Zhou, 2019). For instance, mRNA expression of genes encoding astrocytic glutamate transporters (e.g., SLC1A2 and SLC1A3) and enzymes (e.g., glutamine synthetase) are reduced in the anterior cingulate cortex and dorsolateral prefrontal cortex of patients with MDD (Choudary et al., 2005). These genetic abnormalities would impair astrocytes in their ability to uptake glutamate from the synaptic cleft after neurotransmission, thereby letting the neurotransmitter exert its effects longer, and possibly leading to excitotoxicity (Choudary et al., 2005). Notably, mRNA expression for two specific GABA receptor subunits (GABAAα1 and GABAAβ3) were selectively upregulated, relative to non-suicidal controls, in the anterior cingulate cortex of MDD patients who died by suicide, thus serving as a potential biomarker for suicidality (Choudary et al., 2005). Another biomarker would be the microglial functional state, as microglial priming was shown to correlate with increase suicidal behavior (Gonçalves de Andrade et al., 2022).

### Structural connectivity

Structural abnormalities in MDD largely parallel observations in FC: anatomical projections within the DMN and frontal cortex are markedly disrupted (Korgaonkar et al., 2014; Long et al., 2015; Coloigner et al., 2019). Furthermore, altered SC is consistently observed in frontolimbic networks of patients with MDD, including the prefrontal cortex, anterior cingulate cortex, hippocampus, and amygdala (de Kwaasteniet et al., 2013; Coloigner et al., 2019). As for the directionality of these disruptions, SC within the DMN and frontal cortex are reduced (Korgaonkar et al., 2014). Findings of SC disruptions between the frontal cortex and limbic regions in MDD are more inconsistent, with some studies reporting hyperconnectivity (e.g., Long et al., 2015) and others hypoconnectivity (e.g., Wu et al., 2019). Nonetheless, machine learning algorithms can categorize patients with MDD *versus* controls, using whole-brain SC data, with up to 91.7% accuracy (Fang et al., 2012). When evaluating the data with the machine learning algorithm found most helpful, it was discovered that SC within frontolimbic networks were most informative in identifying MDD brains, suggesting that said networks are a primary SC biomarker of MDD (Fang et al., 2012). This would corroborate clinical symptoms of MDD, as frontolimbic networks are associated with stimulus reward associations (Gleich et al., 2015; Long et al., 2015), emotional regulation (Kebets et al., 2021), and executive functioning (Matsuo et al., 2007). Thus, the aforementioned abnormal connectivity in these networks may help explain the skewed evaluation of reward, negative affect, and poor executive functioning which is common with MDD (Kennedy, 2008). In fact, frontolimbic SC, as measured *via* fractional anisotropy in DTI, positively correlated with the symptoms of anhedonia in patients with MDD (Coloigner et al., 2019). In particular, the strength of white matter connections between the frontal lobes and limbic structures correlated with a self-reported inability to feel pleasure (Coloigner et al., 2019).

In terms of non-neuronal cell mechanisms, evidence suggests that reduced oligodendrocyte density is a potential mechanism for the altered SC in MDD (Hamidi et al., 2004). Relative to controls, amygdala tissue samples from people diagnosed with MDD show reduced oligodendrocyte and total glia density–as indexed *via* cell morphology visualized using Nissl stains (Hamidi et al., 2004). Interestingly, this same study found no difference between MDD samples and controls with respect to astrocytic or microglial densities (these glia subtypes were identified using S-100beta antibody for oligodendrocytes and anti-HLA for microglia), suggesting that the reduction in total glia density within the amygdala was mainly due to a reduction in oligodendrocytes (Hamidi et al., 2004). Indeed, this histological finding may help explain the reduced intrinsic FC identified in the amygdala of patients with MDD (Ramasubbu et al., 2014). Another study using end-point polymerase chain reaction on astrocyte and oligodendrocyte samples from patients with MDD found oligodendrocytes to have significantly shorter telomeres and reduced gene expression of oxidative defense enzymes, relative to time of death and age-matched controls (Szebeni et al., 2014). This finding may provide a cellular mechanism for the reduced oligodendrocyte density found in the brains of patients with MDD (Hamidi et al., 2004), as reduced telomere length and a deficiency in antioxidant enzymes would make these glial cells more susceptible to DNA damage and oxidative stress (Szebeni et al., 2014). In corroboration with the null astrocytic findings of Hamidi et al. (2004), telomere length and antioxidant enzyme levels in astrocytes did not differ between MDD brains and controls (Szebeni et al., 2014).

Microglia and astrocytes do, however, influence the abnormal SC observed in patients with MDD, as mood disorders are influenced by inflammation (and vice versa); in fact, this has led to the development of a field of study termed “affective immunology” (Yang et al., 2020). There is strong evidence to suggest that abnormal immune function and MDD are closely related: firstly, human patients diagnosed with MDD were shown to exhibit greater blood concentrations of proinflammatory cytokines (e.g., IL-6, TNF-α) (Dowlati et al., 2010; Alboni et al., 2016). Meta-analyses on the effects of selective serotonin reuptake inhibitors suggest that these drugs induce their anti-depressant effects, at least in part, by reducing the levels of said peripheral proinflammatory cytokines including the aforementioned IL-6 and TNF-α (Wang et al., 2019), with TNF-α also decreased in the brain. Furthermore, genetic mutations in the genes encoding various cytokines (e.g., IL-1β, IL-6, IL-10, TNF-α, C-reactive protein) have been identified as risk factors for MDD development, and cytokine mRNA expression (especially IL-1β) can be used to help identify patients who will be resistant to traditional MDD pharmacological therapies (Barnes et al., 2017). A growing body of evidence suggests that microglia have a large influence over long-term potentiation (LTP) and long-term depression (LTD) (Innes et al., 2019). For example, microglial fractalkine receptor CX3CR1 stimulation has been hypothesized to stimulate excessive microglial-mediated phagocytosis of synaptic elements, reducing opportunities for LTP (Milior et al., 2016; Innes et al., 2019). Indeed, when exposed to chronic unpredictable stress, CX3CR1 knockout mice have shown greater resilience, relative to control mice, against developing MDD-like symptoms (Corona et al., 2010; Hellwig et al., 2016; Milior et al., 2016; Reshef et al., 2017; Rimmerman et al., 2017). Abnormalities in astrocytic communication *via* gap junctions were also robustly associated with MDD specifically in patients who died *via* suicide (Ernst et al., 2011; Nagy et al., 2017; Tanti et al., 2019). Reduced expression of connexin 30 and 34 genes was observed in the frontal cortex, mediodorsal thalamus, and caudate nucleus of patients with MDD who committed suicide, relative to matched sudden-death controls (Ernst et al., 2011; Nagy et al., 2017). This would suggest that dysfunctional astrocyte-to-astrocyte signaling may be related to MDD suicidality and notably in the frontoparietal networks which show abnormal SC in MDD (Fang et al., 2012; de Kwaasteniet et al., 2013; Long et al., 2015; Coloigner et al., 2019; Wu et al., 2019). Astrocyte-to-oligodendrocyte signaling abnormalities were also implicated in MDD: there was reduced connexin 30 expression localized onto oligodendrocytes in the anterior cingulate cortex of MDD suicide victims, relative to matched sudden-death controls (Tanti et al., 2019). Still, the link between microglial alterations and suicide remains to be elucidated.

### Vascular connectivity

Major depressive disorder and vascular diseases often occur comorbidly, especially in late-life depression, leading to the development of a “vascular depression hypothesis” (Alexopoulos et al., 1997; Menard et al., 2017). In line with the aforementioned disruption of FC and SC in the frontal cortex of patients with MDD, ischemic lesions in prefrontal vascular networks were originally hypothesized to be a central mechanism for the “vascular depression hypothesis” (Alexopoulos et al., 1997). When it comes to late-life MDD, VC and SC share an especially strong relationship: a characteristic feature of vascular depression is the development of white matter lesions, as detected *via* white matter hyperintensities in T2 MRI scans (de Groot et al., 2000; Kumar et al., 2000; Taylor et al., 2013; Rushia et al., 2020). In fact, a study of a large cohort of elderly individuals found that those with severe white matter lesions were 3 to 5 times more likely to present MDD symptoms relative to those with mild/no white matter lesions (de Groot et al., 2000). These white matter lesions are hypothesized to index VC abnormalities, and are thus referred to as “leukoaraiosis” (Coffey et al., 1988). In addition to its influence on SC, the impaired VC often observed in late-life depression has been shown to adversely affect FC in the DMN (Wu et al., 2011). More specifically, resting-state connectivity in the medial prefrontal cortex was found to negatively correlate with the volume of leukoaraiosis in patients with late-life depression (Wu et al., 2011). Based on current hypotheses in MDD (Table 3), more research is to do on the causality of the vascular component in MDD in order to consider therapeutic targeting of these alterations.

## Conclusion

**FIGURE 2:**
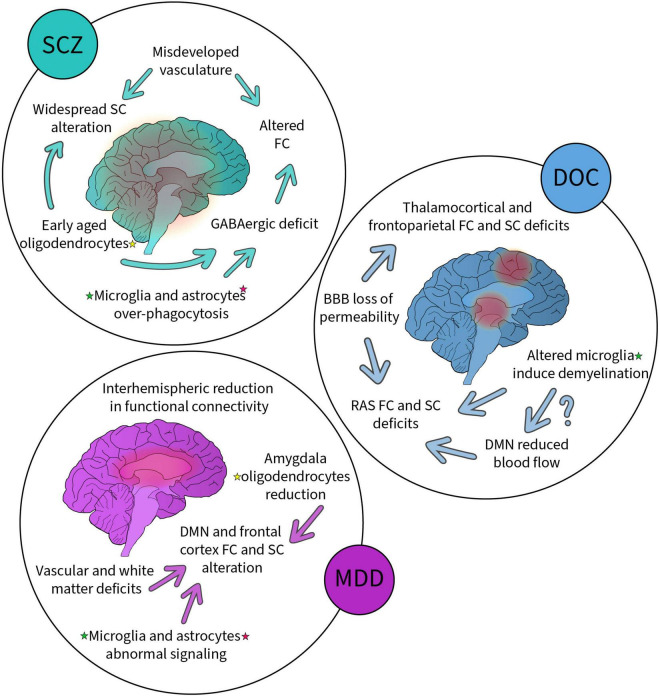
Summary of the alterations to non-neuronal cells, functional, structural, and vascular networks reported in schizophrenia (SCZ), disorders of consciousness (DOC) and major depressive disorder (MDD). Each disorder has been associated with alterations highlighted here. Some alterations are linked (arrows) suggesting possible causality. Altered non-neuronal cells (highlighted with colored stars, yellow for oligodendrocytes, green for microglia and pink for astrocytes) have been found in all these disorders suggesting their key involvement.

As summarized (Figure 2), SCZ shows widespread decrease in SC (Kelly et al., 2018) and abnormalities in FC (Damaraju et al., 2014). SC alterations notably arise from deficits in oligodendrocytes based on genome-wide association analyses (Kerns et al., 2010; Goudriaan et al., 2014). This is in line with other research on glial cells, including oligodendrocytes, which observed signs of accelerated cellular aging in SCZ (Kochunov et al., 2013; Carrier et al., 2021). Microglia and astrocytes are known to over phagocytose in *in vitro* models of SCZ (Sellgren et al., 2019). This pathological feature presents an opportunity for therapeutics not taken yet by the field, as normalization of glial cell functioning might prevent disorder progression. SCZ also has a strong VC component (Carrier et al., 2020). An under-developed vascular network would help explain the alteration in FC and SC, as vasculature is involved in the establishment and maintenance of the GABAergic signaling in the brain (Won et al., 2013; Goudriaan et al., 2014; Hoftman et al., 2015). Proper vascular connectivity is a process that requires the concerted participation of microglia, oligodendrocyte and astrocytes, all essential components of the NVU (Carrier et al., 2020).

DOCs generally present SC and FC impairments in thalamocortical and frontoparietal networks, specifically the RAS, DAN, VAN, and DMN (Wheeler and Malinak, 1989; Adams et al., 2000; Fernández-Espejo et al., 2012; Mecarelli et al., 2019; Tanabe et al., 2020). These abnormalities are found to come mainly from demyelination, notably due to microglia-mediated chronic inflammation (Gosseries et al., 2011; Guldenmund et al., 2016; Bagnato et al., 2017, 2018, 2021), and increased BBB permeability resulting from impaired microglial and astrocytic functioning (Haruwaka et al., 2019; Heithoff et al., 2021). The BBB is at the center of VC alterations after brain injury, but its influence on brain networks remains to be investigated within the context of injuries specific to DOCs. However, patients with DOC present reduced blood flow in frontoparietal networks, including the DMN, which may further explain the aforementioned FC observations (Rosazza et al., 2016; Chennu et al., 2017; Zhang et al., 2018). While glial cells are involved in blood flow modulation (Carrier et al., 2020), their implication in the reduction of DMN blood flow is still to be resolved.

Major depression has been associated with various FC and SC network characteristics, including instability and reduced connectivity of the DMN, hypoconnectivity in frontoparietal control networks, and abnormalities in networks involving the limbic system (Korgaonkar et al., 2014; Long et al., 2015; Kaiser et al., 2016; Ye et al., 2016; Li et al., 2018; Coloigner et al., 2019). Furthermore, patients with MDD demonstrate widespread reduction in interhemispheric FC, relative to controls (Guo et al., 2013b; Wang et al., 2013; Zheng et al., 2022). Microglia and astrocytes are known to participate in MDD pathology, particularly in the reduction of glutamate and GABA (Choudary et al., 2005; Bernard et al., 2011; Medina et al., 2016). The observed reduction of oligodendrocytes in the amygdala of patients with MDD could also explain the reported deficits in limbic SC, correlating with the reduction in white matter (Coloigner et al., 2019). When investigating VC in MDD, a vascular hypothesis emerges, much as for SCZ: namely the hypothesis of “vascular depression” in the elderly (Alexopoulos et al., 1997; Menard et al., 2017; Carrier et al., 2020). Critically, vascular depression is consistently accompanied by white matter lesions and thus impaired SC (de Groot et al., 2000; Kumar et al., 2000; Taylor et al., 2013; Rushia et al., 2020).

It is important to keep in mind the essential physiological role of microglia as immune cells of the brain, astrocytes as central regulators of metabolism and nutrient suppliers, and oligodendrocytes as the main insulator of the CNS (Davalos et al., 2005; Nimmerjahn et al., 2005; Hughes and Appel, 2019; Heithoff et al., 2021). These roles are compromised in many diseases/disorders of the nervous system, especially when persistent inflammation and oxidative stress lead to altered glial cell functioning (Lassmann and van Horssen, 2016; Solleiro-Villavicencio and Rivas-Arancibia, 2018; Chen et al., 2020; Lee et al., 2021b). An important take away with respect to diseases involving FC and VC abnormalities is the therapeutic potential of targeting glial cell neurotransmitter signaling (e.g., glutamate transporters on microglia and astrocytes) and oxidative stress metabolism (Sanacora and Banasr, 2013; Oliveira et al., 2016; Zhou et al., 2019; Zhu et al., 2022). However, further research into the mechanisms by which non-neuronal cells contribute to the brain networks underlying SCZ, DOCs, and MDD is first required. It would be ideal to develop glial pharmacology such that certain cell types and states can be targeted: for instance, specifically targeting microglia (and their pathology-specific states) rather than influencing the functioning of microglia, astrocytes, neurons, and macrophages as a whole with a pharmacological treatment (Šimončičová et al., 2022). Given the complexity and mosaic of mechanisms contributing to these disorders, as well as the individual differences in disorder etiology, it is likely that novel advancements in the treatment of SCZ, DOCs, and MDD could be achieved if network neuroscience expanded beyond typical “neuro-centric” studies (Uher, 2011; Oliveira et al., 2016). This may be particularly true for patients who show treatment-resistance to the current neuron-focused therapies, a prevalent issue, as an estimated 30% of patients being treated for MDD, and 34% of those being treated for SCZ demonstrate treatment-resistance (Rush et al., 2006; Al-Harbi, 2012; Potkin et al., 2020). With respect to DOCs, this quad-partite network neuroscience approach may help with the development of more accurate diagnostic techniques, as common behavioral measures of DOCs are estimated to have a misdiagnosis rate as high as 41% (Schnakers et al., 2009). As mentioned, the field would gain from having more longitudinal studies looking in depth into the pathogenesis of neurodevelopmental disorders. Knowing if a lack of myelination is at play or if accelerated aging is a key mechanism in this disorder would benefit the field as well as prompt to take age in account in each study (Carrier et al., 2020).

## Author contributions

MC was responsible for the review coordination, wrote the abstract, introduction, conclusion, and the section on the NVU while taking care of the overall revision, reference search and formatting of the manuscript, and was also the creator of the figures included in the manuscript. KD wrote the sections on SCZ, DOC, and MDD and prepared the tables. BB wrote the section on microglia-neuron interactions. MD revised and contributed to the theoretical and writing parts of the manuscript. M-ÈT oversaw the outline and revision of the manuscript, as well as contributed significantly to the theoretical, and writing parts of the manuscript. All authors contributed to the article and approved the submitted version.
